# Sodium retention in nephrotic syndrome is independent of the activation of the membrane-anchored serine protease prostasin (CAP1/PRSS8) and its enzymatic activity

**DOI:** 10.1007/s00424-022-02682-y

**Published:** 2022-03-21

**Authors:** Daniel Essigke, Bernhard N. Bohnert, Andrea Janessa, Matthias Wörn, Kingsley Omage, Hubert Kalbacher, Andreas L. Birkenfeld, Thomas H. Bugge, Roman Szabo, Ferruh Artunc

**Affiliations:** 1 Division of Endocrinology, Diabetology and Nephrology, Department of Internal Medicine, University Hospital Tübingen, Otfried-Mueller-Str.10, 72076 Tuebingen, Germany; 2 Institute of Diabetes Research and Metabolic Diseases (IDM) of the Helmholtz Center Munich at the University Tübingen, Tuebingen, Germany; 3 German Center for Diabetes Research (DZD) at the University Tübingen, Tuebingen, Germany; 4 Interfacultary Institute for Biochemistry, Tuebingen, Germany; 5 Proteases and Tissue Remodeling Section, NIDCR, National Institutes of Health, Bethesda, MD USA

**Keywords:** Prostasin, Prss8, Epithelial sodium channel, ENaC, Nephrotic syndrome, Prss8-S238A, Prss8-R44Q

## Abstract

Experimental nephrotic syndrome leads to activation of the epithelial sodium channel (ENaC) by proteolysis and promotes renal sodium retention. The membrane-anchored serine protease prostasin (CAP1/PRSS8) is expressed in the distal nephron and participates in proteolytic ENaC regulation by serving as a scaffold for other serine proteases. However, it is unknown whether prostasin is also involved in ENaC-mediated sodium retention of experimental nephrotic syndrome. In this study, we used genetically modified knock-in mice with *Prss8* mutations abolishing its proteolytic activity (Prss8-S238A) or prostasin activation (Prss8-R44Q) to investigate the development of sodium retention in doxorubicin-induced nephrotic syndrome. Healthy Prss8-S238A and Prss8-R44Q mice had normal ENaC activity as reflected by the natriuretic response to the ENaC blocker triamterene. After doxorubicin injection, all genotypes developed similar proteinuria. In all genotypes, urinary prostasin excretion increased while renal expression was not altered. In nephrotic mice of all genotypes, triamterene response was similarly increased, consistent with ENaC activation. As a consequence, urinary sodium excretion dropped in all genotypes and mice similarly gained body weight by + 25 ± 3% in Prss8-wt, + 20 ± 2% in Prss8-S238A and + 28 ± 3% in Prss8-R44Q mice (*p* = 0.16). In Western blots, expression of fully cleaved α- and γ-ENaC was similarly increased in nephrotic mice of all genotypes. In conclusion, proteolytic ENaC activation and sodium retention in experimental nephrotic syndrome are independent of the activation of prostasin and its enzymatic activity and are consistent with the action of aberrantly filtered serine proteases or proteasuria.

## Introduction

The epithelial sodium channel (ENaC) expressed in the distal nephron is an essential determinant of sodium homeostasis. Among many redundant factors regulating ENaC, channel activation through proteolytic processing by serine proteases is a specific feature of ENaC [24, 27]. Proteolytic activation takes place at specific sites within the extracellular loops of the α- and γ-subunits (but not the β-subunit) and releases inhibitory tracts that induce a conformational change of the channel favoring its open state [2, 23, 24, 28]. Physiologically involved serine proteases are furin, an intracellular serine protease, and prostasin or channel activating protease 1 (CAP1), a glycosylphosphatidylinositol (GPI)-anchored membrane-bound serine protease expressed in the renal tubular system [13, 17, 21]. In *Xenopus laevis* oocytes, co-expression of ENaC with prostasin facilitates proteolytic channel activation by recruitment of an endogenous aprotinin-sensitive serine protease [1, 14]. ENaC activation is preserved when ENaC-expressing oocytes are co-expressed with enzymatically inactive prostasin (Prss8-S238A), indicating an essential scaffold function of prostasin independent from its enzymatic activity [14]. Accordingly, the renal phenotype of Prss8-S238A mutant mice is not different from that of Prss8-wt mice [18]. However, mutation of the activation site of prostasin (zymogen-locked, Prss8-R44Q [16, 17]) leads to impaired proteolytic activation of ENaC in the *Xenopus laevis*-oocyte expression system, probably due to reduced recruitment of an endogenous serine protease [16]. In vivo, healthy Prss8-R44Q mutant mice develop hyperaldosteronism under a low sodium diet and an acquired type 1 pseudo-hypoaldosteronism phenotype upon continuous treatment with the ENaC blocker triamterene [16]. In kidneys from triamterene-treated Prss8-R44Q mice, expression of fully cleaved γ-ENaC failed to increase in contrast to Prss8-wt and Prss8-S238A mice.

In nephrotic syndrome, activation by aberrantly filtered serine proteases or proteasuria is thought to mediate ENaC activation and sodium retention [3, 6]. This coincides with an increased expression of cleavage products of both α- and γ-ENaC in nephrotic mice [11]. Treatment with the ENaC blocker amiloride or the serine protease inhibitor aprotinin prevents ENaC activation and sodium retention in experimental nephrotic syndrome of mice [8, 12, 36]. So far, the exact identity of the essential serine proteases is not known, and there are several serine proteases found in healthy and nephrotic urine [34]. Plasminogen with its active form plasmin is the most abundant serine protease in nephrotic urine and has been proposed to mediate ENaC activation by cleavage of γ-ENaC [30–32]. The same authors also reported that low concentration of plasmin may activate prostasin which would in turn mediate proteolytic ENaC activation [33]. On the other hand, recent work from our group demonstrated that mice lacking urokinase-type plasminogen activator (*Plau*^*−/−*^) or plasminogen (*Plg*^*−/−*^) were not protected from ENaC activation and sodium retention in experimental nephrotic syndrome [8, 36]. Noteworthy, Plg-deficient nephrotic mice were still protected when treated with the serine protease inhibitor aprotinin. A possibility could be that aberrantly filtered serine proteases other than plasmin could mediate ENaC activation indirectly after binding to or activating prostasin which itself is also an aprotinin-sensitive serine protease.

To study the in vivo relevance of prostasin’s role in ENaC-mediated sodium retention in nephrotic syndrome, we studied knock-in mice with enzymatically inactive (Prss8-S238A) or zymogen-locked prostasin (Prss8-R44Q). We report that both genotypes are not protected from proteolytic ENaC activation, indicating that sodium retention occurs independent of prostasin activation and its enzymatic activity.

## Materials and methods

### Mouse studies

Experiments were performed on 3-month-old genetically modified knock-in mice of both sexes carrying either one of two different mutations of Prss8 leading to enzymatic inactivity (Prss8-S238A [26]) or a zymogen-locked state (Prss8-R44Q [17]). Compared to *Prss8* knock-out animals, both mouse strains had a rather mild phenotype with defects in whisker and pelage hair formation (for a photo see Suppl. Figure 1 of our previous study [16]). Imported Prss8-S238A and Prss8-R44Q mice on a mixed background were backcrossed over 6 generations onto a 129 S1/SvImJ background to confer susceptibility to experimental nephrotic syndrome [5, 9]. Genotyping was done using PCR [16]. Mice were kept on a 12:12-h light–dark cycle and fed a standard diet (ssniff, sodium content 0.24% corresponding to 104 µmol/g, Soest, Germany) with tap water ad libitum.

Experimental nephrotic syndrome was induced after a single intravenous injection of doxorubicin (14.6 µg/g body weight [bw], Teva, Germany) as developed by our group [7, 10]. Mice were kept in their normal cages to reduce stress after doxorubicin injection. During the course of nephrotic syndrome, samples of spontaneously voided urine were collected in the morning between 8 and 9 a.m. 2 days before (baseline) and up to 10 days following doxorubicin injection, and daily food and fluid intakes were monitored by weighing the food pellets and the water bottle. Sodium balance was inferred from urinary sodium excretion in relation to food intake and body weight change. To assess ENaC activity, triamterene-stimulated natriuresis was studied before and during avid sodium retention on day 7 and day 8 after induction in a subset of mice. To this end, mice were injected with vehicle (5 µL/g bw injectable water, day 7) and triamterene (10 µg/g bw) on the next day (day 8) to determine urinary sodium excretion during 6 h after injection. Triamterene-sensitive natriuresis was expressed as the ratio from both values. All the animal experiments were conducted according to the National Institutes of Health Guide for the Care and Use of Laboratory Animals and the German law for the welfare of animals, and they were approved by local authorities (Regierungspraesidium Tuebingen, approval number M6/17).

### Quantitation of urinary protease activity against the prostasin cleavage site in γ-ENaC using AMC-coupled peptide substrates

Peptide substrates representing the different cleavage events within the prostasin cleavage site of murine γ-ENaC^180–186^ were synthesized and C-terminally coupled with the fluorophore 7-amino-4-methylcoumarin (AMC) yielding Acetyl (Ac)-FTGR-AMC, Ac-FTGRK-AMC, Ac-FTGRKR-AMC, and Ac-FTGRKRK-AMC as described [35]. To quantitate urinary protease activity, 5 μL mouse urine (1:10 diluted with PBS) was incubated with 5 µL of the AMC-coupled substrates (0.2 mg/mL, 1:10 dilution in PBS) in a total volume of 100 µL in black microtiter plates at 37 °C for 4 h. To test the dynamic range of the assay, trypsin (sequencing grade, Serva, Heidelberg, Germany) was incubated in low and high final concentrations (0.025 and 0.1 mg/mL, respectively). Fluorescence was measured on a microplate reader with excitation/emission at 380/460 nm (gain 80, Tecan Spark 10 M, Germany).

### Laboratory measurements

Urinary creatinine was measured with a colorimetric Jaffé assay (Labor + Technik, Berlin, Germany), urinary protein concentration using the Bradford method (Bio-Rad Laboratories, Munich, Germany) and urinary sodium concentration with flame photometry (Eppendorf EFUX 5057, Hamburg, Germany). Spot urinary protein and sodium concentration were normalized to the urinary creatinine concentration. Plasma urea was measured enzymatically using a colorimetric assay (Labor + Technik, Berlin, Germany). Urinary prostasin excretion and plasma aldosterone concentrations were measured using ELISA kits (Abcam, Cambridge, UK and IBL, Hamburg, Germany). Plasma sodium and potassium were measured using an IL GEM® Premier 3000 blood gas analyzer (Instrumentation Laboratory, Munich, Germany).

### Western blot from kidney tissue of mice

Western blot analysis of prostasin and ENaC expression was performed from a membrane protein preparation of kidney cortex collected under control condition or on the 7th day after induction of nephrotic syndrome when urinary sodium retention concentration dropped below 20 mM, indicating maximal ENaC activation. In addition, we studied kidneys from mice treated with a low sodium diet (C1036, sodium content 0.01% corresponding to 10 µmoL/g) for 4 days. Half the kidney per mouse was sliced, and the cortex was dissected using a scalpel. Homogenization was performed using a Dounce homogenizator in 1 mL lysis buffer containing 250-mM sucrose, 10-mM triethanolamine HCl, 1.6-mM ethanolamine, and 0.5-mM EDTA at pH 7.4 (all Sigma) [37]. During all the preparation steps, aprotinin (40 µg/mL) and a protease inhibitor cocktail (final concentration 0.1 × stock; mini-complete, Roche) was present to avoid ENaC cleavage in vitro. Homogenates were centrifuged at 1000 g for 15 min at 4 °C for removal of the nuclei. Subsequently, the supernatant was centrifuged at 20,000 g for 30 min at 4 °C, and the resulting pellet containing plasma membranes was resuspended in a lysis buffer and diluted to a concentration of 5 mg/mL. Samples were deglycosylated using PNGaseF according to the manufacturer’s instructions (NEB, Ipswich, USA) as previously described [11, 19]. Firstly, samples were denaturated with a glycoprotein denaturing buffer for 10 min at 70 °C. Samples were then incubated with a glycobuffer, NP-40, and PNGaseF for 1 h at 37 °C. Native samples without deglycosylation were boiled in a reduced Laemmli buffer at 70 °C for 10 min. Subsequently, 25 µg of sample was loaded on 8% (prostasin, γ-ENaC) or 4–15% gradient (α- and β-ENaC) polyacrylamide gels for electrophoresis. Recombinant murine prostasin was used as a positive control (amino acids 30–289, R&D systems). After SDS-PAGE under reducing conditions, prostasin, and ENaC subunits were probed with affinity-purified rabbit antibodies against murine α- or β-ENaC (custom made by Dr. Pineda, Berlin, Germany), rat γ-ENaC (SPC-405, Stressmarq, Viktoria, Canada) [8, 25] or prostasin (15,527–1-AP, proteintech) overnight at 4 °C after 1:1000 dilution in a blocking buffer (Licor, Lincoln, USA). Signals were detected with a fluorescent secondary antibody labeled with IRDye 800CW and a fluorescence scanner (Licor Odyssey, Lincoln, USA). After detection of α-ENaC, the membranes were stripped with a stripping buffer (Licor) and re-probed for detection of β-ENaC. For loading control, total protein was measured using revert total protein stain (Licor, Lincoln, USA). For densitometry, ENaC signals were normalized for total protein signal of the entire lane using Empiria Studio version 2.2 (Licor, Lincoln, USA).

### Statistical analysis

Data are provided as means with SEM. Data were tested for normality with the Kolmogorov–Smirnov-Test, D’Agostino and Pearson omnibus normality test and Shapiro–Wilk-Test. Variances were tested using the Bartlett’s test for equal variances. Accordingly, data were tested for significance with parametric or non-parametric ANOVA followed by Dunnett’s, Dunn’s, or Tukey’s multiple comparison post hoc test, paired or unpaired Student’s *t*-test, or Mann–Whitney *U*-test where applicable using GraphPad Prism 9, GraphPad Software (San Diego, CA, www.graphpad.com). Densitometric analysis of western blots was done using Empiria Studio version 2.2 (Licor, Lincoln, USA). A p value < 0.05 at two-tailed testing was considered statistically significant.

## Results

### Expression of prostasin in experimental nephrotic syndrome

**Fig. 1:**
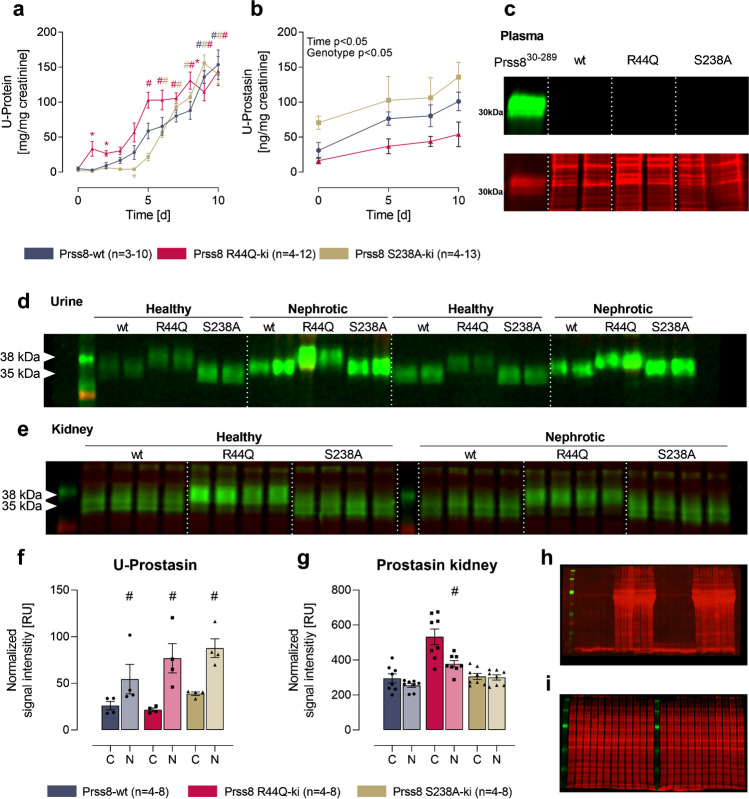
Induction of nephrotic syndrome and expression of prostasin in Prss8-wt, Prss8-S238A, and Prss8-R44Q mice. **a** Course of proteinuria after injection of doxorubicin at day 0. **b** Course of the urinary excretion of prostasin, measured with ELISA. **c** Western blot for expression of wild-type and mutant prostasin in the plasma with the loading control below. Recombinant truncated murine prostasin (amino acids 30–289, predicted mass 28 kDa) served as positive control. **d–e** Western blot for expression of wild-type and mutant prostasin in the urine (**d**) and in kidney lysates (**e**). The dashed white line is only for optical discrimination, it is one blot each, without cutting. **f–g**. Densitometric analysis of prostasin expression in urine (*n* = 4) and kidney (*n* = 8). **h–i** Total protein stain for loading control of the blots shown in d (**h**) and e (**i**). # indicates significant difference between healthy and nephrotic state, * indicates significant difference to the wildtype

To determine whether prostasin activation or prostasin activity is essential for proteolytic ENaC activation and sodium retention in experimental nephrotic syndrome, we administered doxorubicin to Prss8-wildtype mice and those with point mutations of Prss8 at the activation site (Prss8-R44Q) or the active site (Prss8-S238A). Following a single doxorubicin injection all the genotypes developed similar nephrotic-range proteinuria (Fig. 1a). This was paralleled by an increase in the urinary prostasin excretion as quantitated by ELISA (Fig. 1b). In Western blot analyses from plasma samples, prostasin expression was absent in all the genotypes (Fig. 1c), whereas in urine samples, prostasin was detected at 35 kDa in Prss8-wt and Prss8-S238A mice (Fig. 1d, h), corresponding to the heavy chain of activated prostasin after dissociation of the disulfide bond under reducing conditions. In Prss8-R44Q mice with zymogen-locked prostasin, the two-chain form of full-length prostasin was detected at 38 kDa. In nephrotic samples, urinary excretion of prostasin was increased across all the genotypes (Fig. 1f). Western blot of kidney tissue demonstrated that the prostasin expression was not different across the genotypes under control conditions and did not increase after induction of nephrotic syndrome (Fig. 1e, g–i).

### Urinary protease activity against a peptide substrate containing the prostasin cleavage site of γ-ENaC

**Fig. 2:**
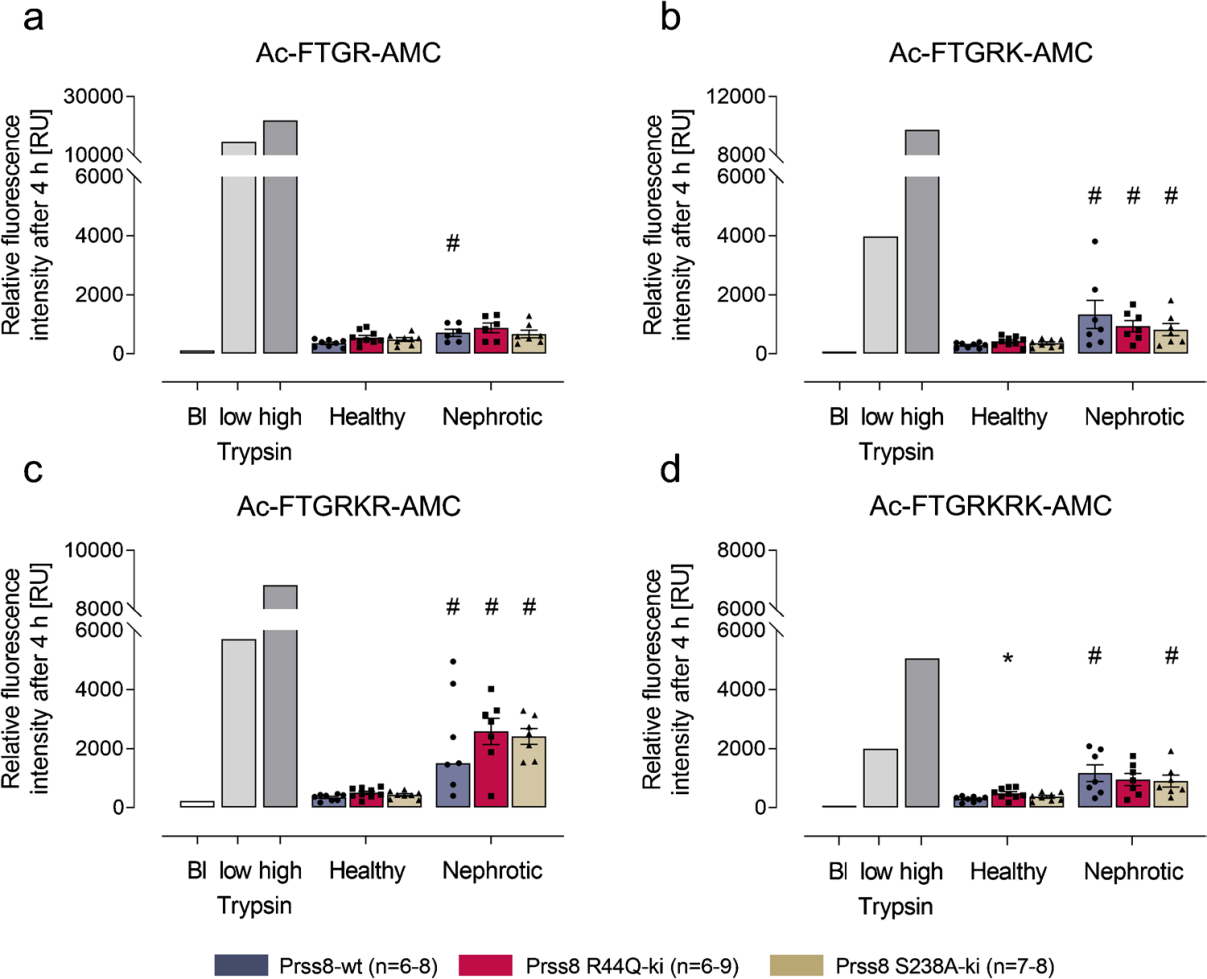
Urinary protease activity against a peptide substrate containing the prostasin cleavage site of γ-ENaC. Relative fluorescence signal reflecting amidolytic activity after 4 h incubation against Ac-FTGR-AMC (**a**), Ac-FTGRK-AMC (**b**), Ac-FTGRKR-AMC (**c**), and Ac-FTGRKRK-AMC (**d**) in urine samples from healthy or nephrotic Prss8-wt, Prss8-R44Q, and Prss8-S238A mice. Trypsin was used in two concentrations (0.025 mg/mL and 0.1 mg/mL, respectively) to determine the dynamic range of the assay. # indicates significant difference between healthy and nephrotic state. Abbreviations: *Bl* blank

The murine prostasin cleavage site corresponds to a polybasic tract at γ-ENaC^183–186^ with the sequence RKRK [13]. However, the exact cleavage pattern of this tract by prostasin is not clear. To quantitate urinary prostasin activity and to capture all cleavage events within the polybasic tract, we synthesized fluorogenic substrates of different lengths and incubated them with urine samples from healthy and nephrotic mice. Amidolytic activity against Ac-FTGR-AMC (Fig. 2a), Ac-FTGRK-AMC (Fig. 2b), Ac-FTGRKR-AMC (Fig. 2c), and Ac-FTGRKRK-AMC (Fig. 2d) was very low in healthy Prss8-wt mice. The activity in urine samples from Prss8 mutant mice was not lower, probably missing the contribution of urinary prostasin activity to the overall signal. In contrast, amidolytic activity was significantly increased in urine samples from nephrotic Prss8-wt mice as well as in samples from nephrotic Prss8 mutant mice, indicating proteasuria. Again, there was no significant difference between the genotypes.

### Sodium handling in nephrotic Prss8-S238A and Prss8-R44Q mutant mice

**Fig. 3:**
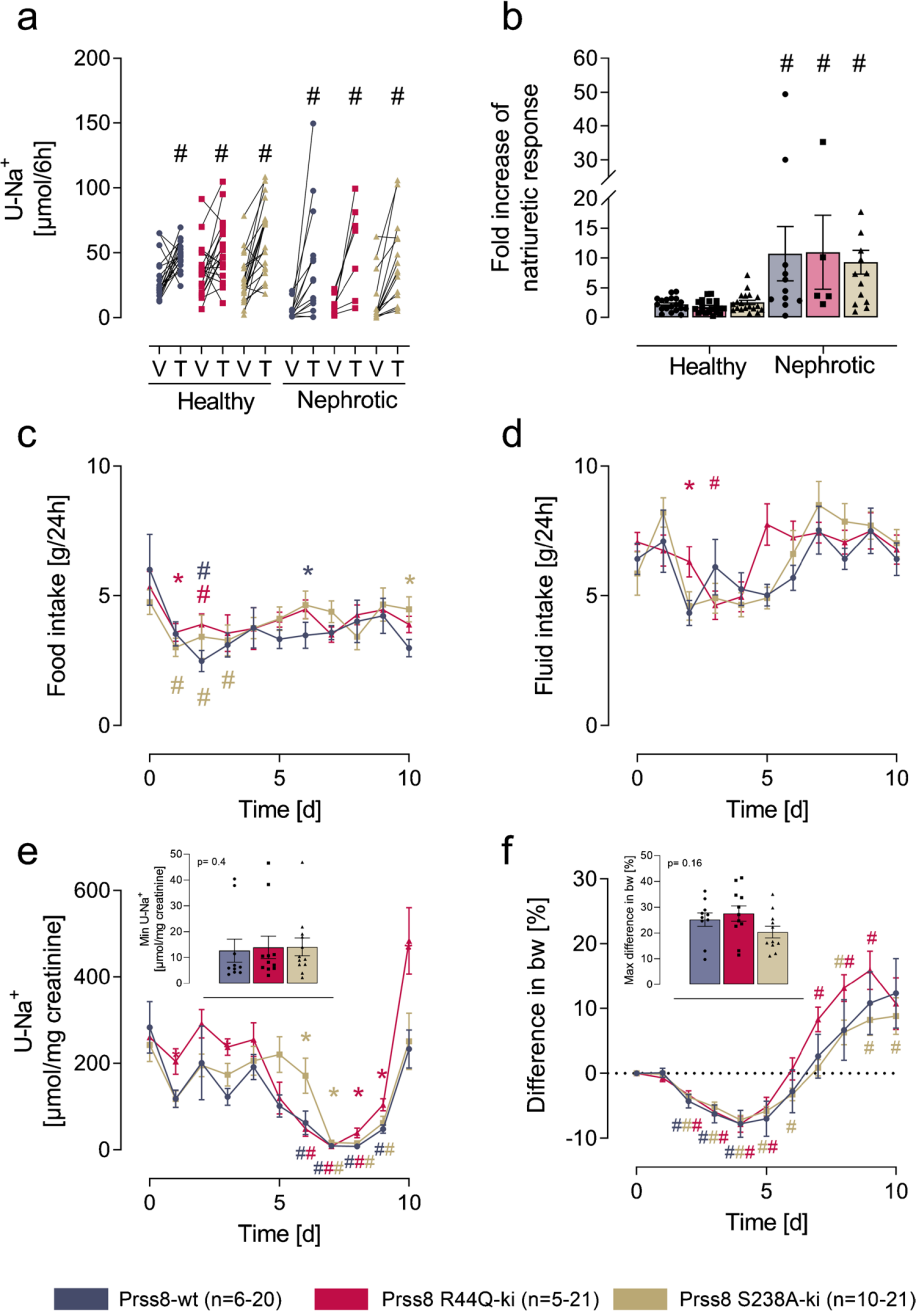
Activation of ENaC in Prss8-wt, Prss8-S238A, and Prss8-R44Q mice before and after induction of nephrotic syndrome. **a** Natriuretic response to the acute administration of the ENaC inhibitor triamterene (T, 10 µg/g) or vehicle injection (V, injectable water, 5 µL/g). **b** Fold-increase of the natriuretic response after triamterene administration. **c–f** Course of food and fluid intake, urinary sodium excretion in spot urine samples, and body weight taken in the morning after induction of nephrotic syndrome. Inset in **g** and **f** depict the minimal urinary sodium excretion and maximal body weight gain, both reflecting maximal ENaC activation. Abbreviations: V vehicle T triamterene. # indicates significant difference between healthy and nephrotic state, * indicates significant difference between the genotypes

The natriuretic response to triamterene (10 µg/g bw i.p.) was determined to assess ENaC activity in Prss8-wt, Prss8-S238A, and Prss8-R44Q mice. Baseline natriuresis was determined from injection of vehicle (injectable water, 5 µL/g bw). As shown in Fig. 3a, this response is similar in all the genotypes before induction of nephrotic syndrome, indicating similar ENaC activity. After induction of nephrotic syndrome, natriuretic response increased significantly in all genotypes reaching similar values. ENaC activation in nephrotic mice is most evident when the triamterene sensitive natriuresis (ratio of natriuresis between vehicle and triamterene) is calculated (Fig. 3b), corresponding to the slope in Fig. 3a. This factor was not different between the genotypes (*p* = 0.84).

After doxorubicin injection, food and fluid intakes were transiently reduced and returned to normal values thereafter (Fig. 3c, d). Urinary sodium excretion was similar in all the genotypes at baseline and after onset of proteinuria on day 5, daily urinary sodium excretion decreased in all the genotypes despite food intake had normalized (Fig. 3e). Between days 7 and 9, urinary sodium excretion fell to minimal values of 13 ± 4 µmoL/mg creatinine in Prss8-wt, 14 ± 3 µmoL/mg creatinine in Prss8-S238A and 14 ± 4 µmoL/mg creatinine in Prss8-R44Q mice (*p* = 0.44, inset Fig. 3g), indicating an almost sodium-free urine. In the first days following doxorubicin injection, body weight decreased to the same extent in all three genotypes due to inappetence (Fig. 3c, d). Thereafter, body weight steeply increased in all the genotypes and mice developed ascites (Fig. 3f). Maximal body weight gain was + 25 ± 3% in Prss8-wt, + 20 ± 2% in Prss8-S238A, and + 28 ± 3% in Prss8-R44Q mice which was not significantly different (*p* = 0.16, inset Fig. 3h).

Table 1 depicts the alterations of the plasma concentrations of electrolytes, hematocrit, and hemoglobin concentration as determined using a blood gas analyzer. There were no differences in any parameter in healthy Prss8-wt, Prss8-S238A, and Prss8-R44Q mice. In the nephrotic state, plasma potassium concentrations were significantly increased compared to baseline in Prss8-wt and Prss8-R44Q mice. This has been analogously shown in nephrotic rats [38] and mice [15].

**Table 1:** Plasma parameters obtained from venous blood gas analysis as well as plasma urea and aldosterone concentration in Prss8-wt, Prss8-S238A, and Prss8-R44Q mice before and 10 days after induction of nephrotic syndrome

	Healthy			Nephrotic		
	Prss8-wt	Prss8-R44Q	Prss8-S238A	Prss8-wt	Prss8-R44Q	Prss8-S238A
Venous pH	7.26 ± 0.01	7.29 ± 0.01	7.29 ± 0.01	7.29 ± 0.01	7.31 ± 0.01	7.29 ± 0.01
std HCO_3_^−^, mM	21 ± 1	22 ± 1	22 ± 1	24 ± 1^#^	26 ± 1^#^	23 ± 1
Na^+^, mM	149 ± 1	149 ± 1	149 ± 1	142 ± 2^#^	147 ± 2^#^	145 ± 1^#^
K^+^, mM	4.4 ± 0.1	4.0 ± 0.1	4.3 ± 0.1	5.0 ± 0.2^#^	5.0 ± 0.1^#^	4.5 ± 0.2
Ca^++^, mM	1.1 ± 0.02	1.0 ± 0.03*	1.1 ± 0.02	1.1 ± 0.02^#^	1.1 ± 0.02 ^#^	1.1 ± 0.02
Hct, %	45.9 ± 1	45.8 ± 1	46.5 ± 1	41.1 ± 3	38.2 ± 4^#^	40.5 ± 4
cHb, g/dL	15.7 ± 0.5	15.3 ± 0.3	15.4 ± 0.4	13.6 ± 0.9	13.8 ± 0.5^#^	14.6 ± 0.5
Urea, mg/dL	44 ± 7	52 ± 9	48 ± 6	119 ± 30^#^	101 ± 17	61 ± 9
Aldosterone, pg/mL	219 ± 32	123 ± 17	217 ± 36	269 ± 71	230 ± 45	232 ± 47

Arithmetic means ± SEM (*n* = 6–11 each)

*std* standard, *Hct* hematocrit, *cHb* calculated hemoglobin concentration

^#^Significant difference between uninduced and nephrotic mice of the same genotype

^*^Significant difference compared to the wildtype

### Expression of ENaC subunits and proteolytic processing in nephrotic Prss8 mutant mice

**Fig. 4:**
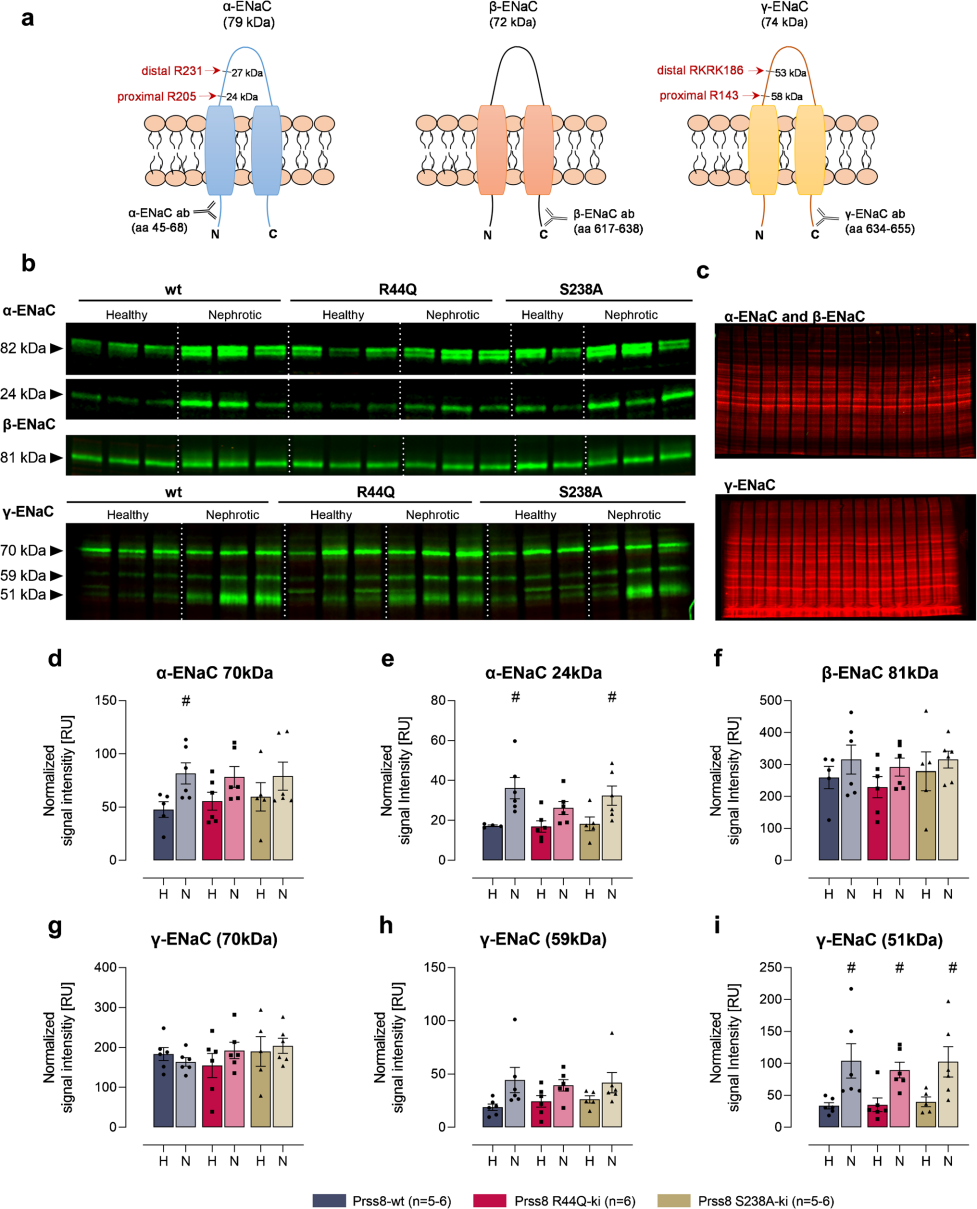
Expression of ENaC subunits and proteolytic processing in Prss8-wt, Prss8-S238A, and Prss8-R44Q mice before and after induction of nephrotic syndrome. **a** Localization of the immunogenic sequences of the used antibodies against murine α-, β- and γ-ENaC. In α- and γ-ENaC, the proximal and distal cleavage sites (designated from the N-terminus, respectively) are depicted. The antibody against N-terminal α-ENaC is supposed to detect full-length α-ENaC at 79 kDa (699 aa) and two N-terminal fragments with a mass of 27 kDa (231 aa), and 24 kDa (205 aa). The antibody against C-terminal β-ENaC is supposed to detect full-length β-ENaC at 72 kDa (638 aa). The antibody against C-terminal γ-ENaC is supposed to detect full-length γ-ENaC at 74 kDa (655 aa) and C-terminal fragments with a mass of 58 kDa (512 aa) after proximal cleavage and at 53 kDa (469 aa) after distal cleavage, respectively. Mass values are calculated from the amino acid sequences (omitting any N-glycosylations). **b** Representative Western blots showing the expression of α-, β- and γ-ENaC in a plasma membrane preparation of kidney cortex lysates before (healthy) and after induction (nephrotic) of nephrotic syndrome. Note that the samples were deglycosylated before analyzing expression of γ-ENaC and its cleavage products [19]. The white line is only for optical discrimination, it is one blot each, no vertical cutting. **c** Total protein stain as a loading control. **d–i** Densitometry of the obtained bands normalized for total protein content of each lane (*n* = 5–6 each). # indicates significant difference between healthy and nephrotic state

In kidney cortex lysates, Western blot analyses identified two bands for α-ENaC at 82 and 24 kDa corresponding to full-length and a cleavage product after distal cleavage (designated from the N-terminus; Fig. 4a–c). For β-ENaC, there was only a single band at 81 kDa corresponding to the full-length subunit which is not proteolytically processed (Fig. 4a–c). For γ-ENaC there were three bands in deglycosylated samples at 70, 59, and 51 kDa (Fig. 4a–c) corresponding to full-length, proximally and distally cleaved fragments, respectively [19]. Specificity of these bands had been demonstrated by application of the immunogenic peptide and recombinantly expressed ENaC subunits as shown elsewhere [11]. In healthy Prss8-wt, Prss8-R44Q, and Prss8-S238A mice, there was no significant difference in the expression of any ENaC subunit. After induction of nephrotic syndrome, the expression of full-length α-ENaC was increased in Prss8-wt mice, an effect not reaching significance in Prss8 mutant mice (Fig. 4d). In all the genotypes, expression of full-length β- and γ-ENaC was not altered (Fig. 4f, g). In nephrotic Prss8-wt, Prss8-R44Q, and Prss8-S238A mice, the expression of the cleavage fragments of α-ENaC at 24 kDa and that of γ-ENaC at 51 kDa, respectively, were found to be significantly increased, indicating proteolytic ENaC activation at both subunits (Fig. 4e, h, i). However, there was no difference between the genotypes.

### Expression of ENaC subunits and proteolytic processing in Prss8 mutant mice after exposure to a low-sodium diet

**Fig. 5:**
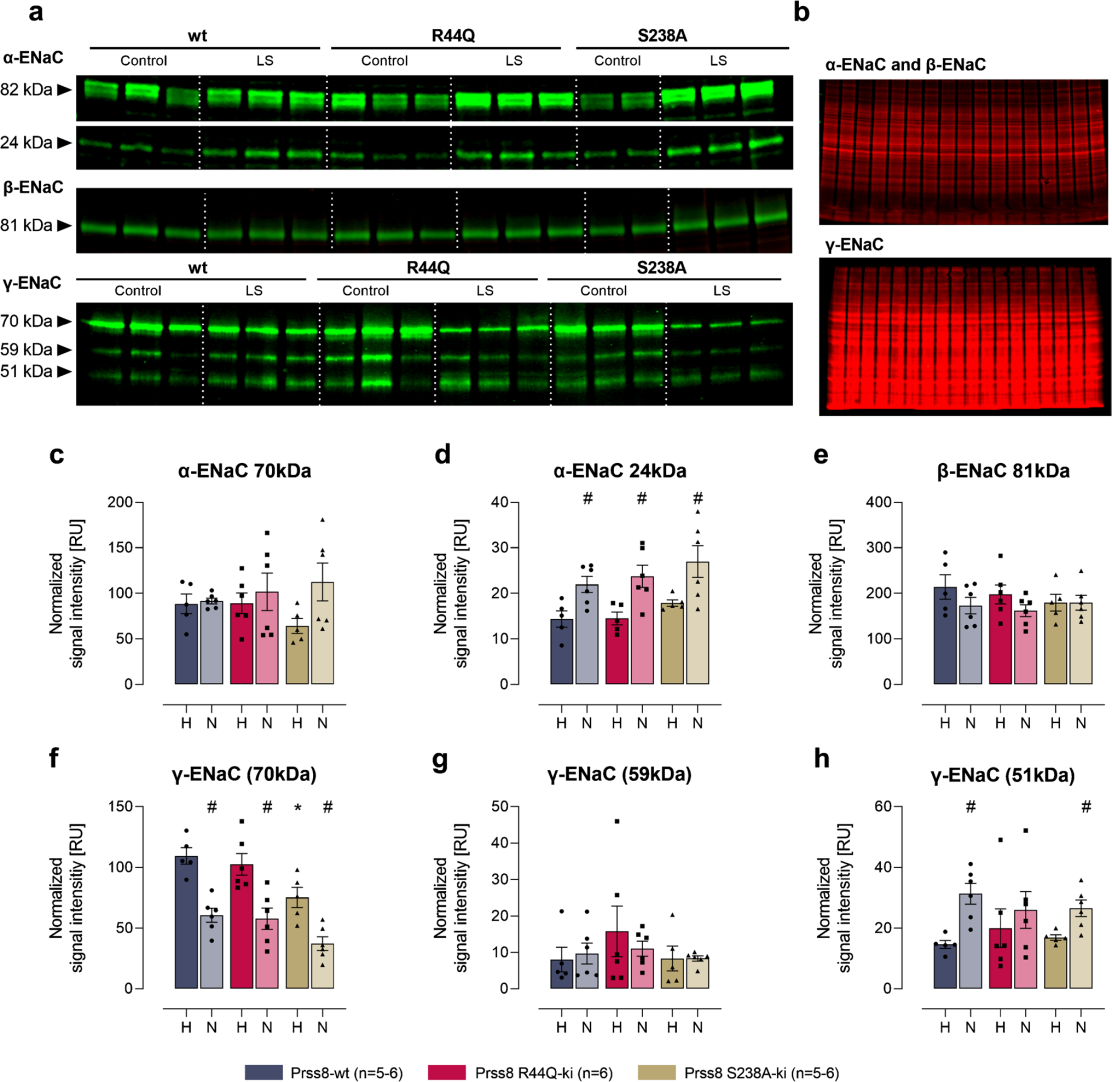
Expression of ENaC subunits and proteolytic processing in Prss8-wt, Prss8-S238A, and Prss8-R44Q mice before and after exposure to a low sodium diet. **a** Representative Western blots showing the expression of α-, β- and γ-ENaC in a plasma membrane preparation of kidney cortex lysates under a control and low sodium (LS) diet. Note that the samples were deglycosylated before analyzing expression of γ-ENaC and its cleavage products [19]. The white line is only for optical discrimination, it is one blot each, no cutting, no cropping. **b** Total protein stain as a loading control. **c–h** Densitometry of the obtained bands normalized for total protein content of each lane (*n* = 5–6 each). # indicates significant difference between healthy and nephrotic state, * indicates significant difference between the genotypes (tested using two-way ANOVA)

To compare the obtained results with the role of prostasin during proteolytic processing of ENaC during sodium restriction and to test the sensitivity of the WB method, we repeated the same WB analyses with mice subjected to a low-sodium diet. In Prss8-wt mice and Prss8-S238A mice, expression of full-length α- and β-ENaC was not altered (Fig. 5a–c, e). The expression of full-length γ-ENaC decreased significantly in all the genotypes (Fig. 5f). In Prss8-wt and Prss8-S238A mice under a low-sodium diet, the expression of the cleavage fragments of α-ENaC at 24 kDa and that of γ-ENaC at 51 kDa were significantly increased, indicating proteolytic ENaC activation at both subunits at the distal cleavage site (from the N-terminus, Fig. 5d, h). In Prss8-R44Q mice treated with a low-sodium diet, the expression of the cleavage fragment of α-ENaC at 24 kDa was similarly increased (Fig. 5d). The expression of furin-cleaved at 59 kDa and fully cleaved γ-ENaC at 51 kDa did not increase to the same extent in Prss8-R44Q mice, suggesting impaired proteolytic ENaC activation as previously described [16].

## Discussion

This study demonstrates that mutated prostasin leading to a zymogen-locked (Prss8-R44Q) or enzymatically inactive (Prss8-S238A) state is dispensable for proteolytic ENaC activation and sodium retention in experimental nephrotic syndrome. These results suggest that sodium retention occurs independent of prostasin activation that is impaired in Prss8-R44Q-mutant mice but preserved in Prss8-S238A-mutant mice. Moreover, enzymatic activity of prostasin which is impaired in both mutants seems also not to be required for proteolytic activation of ENaC in nephrotic syndrome. This in turn suggests that proteolytic ENaC activation in the nephrotic model is accomplished by aberrantly filtered serine proteases that cleave γ-ENaC directly from the tubular lumen and override the effect of serine proteases of the plasma membrane. This is supported by the finding of increased proteolytic activity against peptide substrates containing the sequence of the prostasin cleavage site (RKRK). In contrast, in mice exposed to a low sodium diet proteolytic processing of γ-ENaC tended to be incomplete in Prss8-R44Q mice but was preserved in Prss8-S238A mice, consistent with an impaired recruitment of an endogenously active protease. This finding has been similarly found in Prss8-R44Q mice during prolonged ENaC inhibition by pharmacological treatment with triamterene [16, 29].

It must be emphasized that the results only apply to the prostasin mutants Prss8-R44Q and Prss8-S238A, and there is the theoretical possibility that prostasin might interact with aberrantly filtered serine proteases involving other parts of prostasin. This possibility could only be excluded using prostasin-deficient mice which are, however, not viable [22] or using mice with conditional deletion of prostasin in the kidney. Indeed, in M1 cells derived from cortical collecting duct, there is evidence that prostasin is essential to mediate proteolytic ENaC activation [33]. In these cells, prostasin was found to be co-expressed with ENaC and to recruit the serine protease plasmin to the plasma membrane. Exposure to both nephrotic urine and plasmin stimulated inward currents in these cells which were strongly reduced after transection with prostasin siRNA [33]. In addition, prostasin knockdown attenuated plasmin-induced cleavage of γ-ENaC, indicating an essential role of prostasin in plasmin-induced ENaC activation in vitro. The study, however, did not address a molecular basis for the interaction of prostasin with plasmin, e.g., if the activation site at R44 or enzymatic activity based on S238 was required. Our study was inspired by these in vitro findings and aimed at clarifying the relevance of prostasin for the proteolytic ENaC activation in nephrotic syndrome in vivo. To date, our group has evaluated several serine proteases such as urokinase-type plasminogen activator (encoded by *Plau*), plasminogen (*Plg*), plasma kallikrein (*Klkb1*) or factor VII activating protease (*Habp2*) with regard to their relevance for proteolytic ENaC activation in experimental nephrotic syndrome [4, 8, 20, 36]. All of these activate ENaC in the *Xenopus laevis*-oocyte expression system by cleavage of the γ-subunit as evidenced by Western blot of cell surface expressed ENaC. However, mice with constitutive deletion of any of these genes mentioned above were not protected from proteolytic ENaC activation and sodium retention in experimental nephrotic syndrome. Our group has characterized proteases in nephrotic urine from humans and mice and detected several candidates that could mediate ENaC activation in nephrotic syndrome [34]. In that study, prostasin was detected in healthy urine samples analogous to other renally expressed serine proteases such as uPA. In contrast, nephrotic urine contained high molecular weight serine proteases from the coagulation and complement system. To prove an essential role of any of these candidates in vivo, knockout models are indispensable to validate in vitro data that can be misleading.

The renal expression of prostasin in the kidney was not altered in mice of all the genotypes under control conditions and in nephrotic syndrome. Interestingly, urinary prostasin excretion was increased in nephrotic mice, and in Western blot analyses, urinary prostasin had the exact molecular weight as observed in kidney lysates. Thus, this finding can only be explained by increased shedding of prostasin into the urinary space. The possibility of aberrant filtration in this mouse model is excluded by the fact that prostasin does not circulate in a soluble form and was accordingly not detected in the plasma by Western blot. Although the exact mechanisms of urinary prostasin shedding in nephrotic mice was not addressed, it could be related to increased overall serine protease activity in nephrotic syndrome [34], presumably leading to prostasin cleavage off its GPI-anchor.

In conclusion, this study demonstrates that proteolytic ENaC activation and sodium retention in experimental nephrotic syndrome are independent of activation of the membrane-anchored serine protease prostasin and its enzymatic activity.

## Data Availability

The data that support the findings of this study are available from the corresponding author upon reasonable request.
